# National Health Service maternity charging and migrant women: an unjust policy that fails on its own terms

**DOI:** 10.1093/eurpub/ckag129

**Published:** 2026-07-21

**Authors:** Ahmed Hamed Elnady

**Affiliations:** Institute for Global Health, University College London, London, United Kingdom

In the United Kingdom, migrant women without indefinite leave to remain, including undocumented individuals, refused asylum-seekers, and unidentified victims of trafficking, are required to pay for National Health Service (NHS) maternity care [1, 2]. The Immigration Act 2014 restricted entitlement to free secondary NHS care to those deemed ‘ordinarily resident’, while temporary migrants must pay an Immigration Health Surcharge, now £1035 per year, upfront for the duration of their visa [1]. Those without valid immigration status fall outside both provisions and are charged on a per-treatment basis at 150% of the standard NHS tariff. A straightforward vaginal birth can generate an invoice exceeding £6500, with complications adding substantially more [1]. Although maternity care is classified as ‘immediately necessary’ and should never be withheld, women remain fully liable for the cost. Failure to settle the debt within 2 months may trigger a referral to the Home Office, with consequences for future immigration applications or re-entry to the UK [2, 3]. This viewpoint argues that the policy is not only ethically indefensible but also, according to available evidence, economically counterproductive, and that reform is urgently needed.

A substantial body of evidence indicates that charging may deter many migrant women from seeking timely antenatal care, contributing to delays that carry serious risks for maternal and neonatal health. A 2023 systematic review of the impact of NHS charging regulations on healthcare access among migrants in England identified fear of charges and associated immigration enforcement as a major barrier, particularly for maternity services [4]. The review noted that charging created a climate in which women delayed or avoided seeking care, even when entitled to exemptions. Data from Doctors of the World (DOTW), who provide healthcare to populations excluded from mainstream services, illustrate the scale of late presentation among affected women. Of those accessing DOTW services between 2017 and 2021, 81% of pregnant women had their first antenatal appointment beyond the recommended 10 weeks of gestation, and 45% did not access care until after 16 weeks, compared with only 10% of women nationally [5]. Over a third of these women had been charged for NHS care, often inappropriately [5]. Some women presented only at the point of delivery, forgoing antenatal monitoring entirely [2, 5].

The clinical significance of delayed initiation of antenatal care is well established, with such delays associated with higher rates of preterm birth, low birth weight, undiagnosed pre-eclampsia, and preventable maternal death [1, 6]. These findings must be read alongside the persistent inequalities in maternal outcomes documented by MBRRACE-UK. The 2024 report found that women from Black ethnic backgrounds were almost three times as likely to die during or shortly after pregnancy compared with White women, while women living in the most deprived areas faced more than double the mortality rate of those in the least deprived areas [7]. The same report included a dedicated morbidity enquiry into the care of migrant women who had arrived in the UK <2 years before pregnancy, revealing significant deficiencies in care quality, language support, and access to antenatal pathways [7].

These data describe the broader landscape of vulnerability within which charging operates. Within that landscape, charging adds a financial deterrent on top of existing linguistic, cultural, and administrative barriers, and is therefore likely to deepen the disadvantage migrant women already face. Qualitative research indicates that receiving large NHS invoices generates significant psychological distress, which may compound the elevated rates of postnatal depression observed among migrant women [2]. Because severe maternal stress during pregnancy is itself an independent risk factor for adverse obstetric outcomes, a policy intended to recover costs can contribute to additional health burdens [1]. These harms are not incidental; they reflect deeper structural patterns of inequality. The categories that determine entitlement to free NHS care, ‘ordinarily resident’, ‘overseas visitor’, and ‘chargeable migrant’, are socially constructed classifications that produce and sustain inequalities by treating contingent social distinctions as fixed bases for differential access to resources. Moreover, the policy is uniquely gendered. Although pregnancy results from the actions of two individuals, only the woman bears the financial liability, and, if the debt remains unpaid, the immigration consequences. Male partners are entirely absolved of responsibility [1]. When gender intersects with undocumented immigration status, poverty, language barriers, and racialization, the result is a compounding of disadvantage, which intersectional analysis would recognize as overlapping systems of oppression that the policy actively deepens rather than merely reflects [2].

Proponents of charging frame it as necessary cost recovery, yet the available evidence indicates that the system is administratively inefficient and that its economic rationale is weak. Evidence submitted to the UK Parliament by Maternity Action revealed that one in three NHS hospital trusts spent more on employing staff to administer the overseas charging system than they recovered in payments, and that 28% of trusts consumed over 60% of recovered income merely to operate the billing process [3]. The National Audit Office acknowledged that the Department of Health lacked a clear understanding of the administrative costs trusts incur in seeking to recover these charges [3], and that trusts recover only around half of what they charge directly to patients, while debt collection via private agencies further erodes any net revenue [8].

No published study sets the full administrative and downstream health costs of charging against the income it generates; international bodies have in fact called for exactly this kind of cost-and-benefit monitoring of migrant exclusion to be established [9]. On the evidence available, charging is administratively inefficient and its net fiscal benefit is doubtful.

A separate line of evidence concerns the downstream costs of deterred care. A 2024 systematic review of interventions to improve perinatal care for migrant women found that removing financial barriers to maternity services improves health outcomes and can reduce overall healthcare expenditure, since early access to antenatal care prevents complications that are more costly to manage at later stages [6].

The case for reform is compelling across clinical, ethical, and economic dimensions. A wide range of professional bodies, including several of the Royal Colleges, have called for NHS maternity charging to be suspended [4]. The specific measures required are clear. First, charging for all maternity care, across antenatal, intrapartum, and postnatal services, should be suspended for chargeable migrant women, along with the data-sharing and debt-reporting arrangements that link unpaid NHS debt to immigration enforcement, which serve as a powerful deterrent to care-seeking. Second, primary care registration must be consistently upheld regardless of immigration status or documentation, and all women should receive clear, translated information about their healthcare entitlements and any charging rules that apply to them.

If the United Kingdom is serious about reducing maternal health inequalities, a commitment repeatedly stated in national policy and underscored by each successive MBRRACE-UK report, then continuing to charge the most vulnerable women for essential pregnancy care is incoherent.

The UK case illustrates a wider European dilemma, as legal entitlements to publicly funded care for undocumented migrants vary considerably across Europe, and many countries restrict access even to essential and emergency services [9]. A systematic review of asylum seekers and undocumented migrants in Europe found worse maternal and perinatal outcomes among these populations, including elevated risks of preterm birth and low birth weight [10]. The UK experience therefore offers a transferable lesson. Where entitlement rules and immigration enforcement extend into maternity care, they risk deterring the women most in need of it, while the administrative effort of recovering charges may absorb much of what it collects. The international call to extend coverage to the whole resident population, and to build the data systems needed to measure the costs and benefits of exclusion, applies directly to any high-income system weighing entitlement restrictions against maternal health [9]. Universal, non-discriminatory access to maternity care is the ethically necessary course, and likely the more cost-effective one as well.

Conflict of interest: None declared.

## Data Availability

There are no new data associated with this viewpoint. NHS charging for maternity care deters many migrant women from accessing timely antenatal services, with 81% of women in one study booking beyond 10 weeks and 45% beyond 16 weeks of gestation, compared with around 10% nationally. One in three NHS trusts spends more on administering the overseas charging system than it recovers in payments, undermining the policy’s own economic rationale. Suspending maternity charges and associated immigration data-sharing is likely to improve maternal and neonatal outcomes and reduce overall healthcare costs.
